# Retinal astrocytic hamartoma in tuberous sclerosis complex in an elderly person: a case report

**DOI:** 10.1186/s12886-018-0991-z

**Published:** 2018-12-12

**Authors:** Xiuhong Qin, Yuan Tao, Zhenzhen Zhang

**Affiliations:** 1 Department of Ophthalmology, First Affiliated Hospital of Dalian Medical University, No 222 Zhongshan Road, Xigang Strict, Dalian, 116011 Liaoning Province China; 2 Department of Ophthalmology, the Ninth People’s Hospital, Shanghai Jiao Tong University, No 639 ZhiZaoJu Road, Shanghai, 200011 China

**Keywords:** Tuberous sclerosis complex, TSC, Retinal astrocytic hamartoma, RAH, Astrocytes, Optical coherence tomography, Ophthalmic examination

## Abstract

**Background:**

Spectral domain optical coherence tomography (SD-OCT) is proposed as a way of predicting the development and likelihood of retinal astrocytic hamartoma (RAH) in tuberous sclerosis complex (TSC) in elderly patients.

**Case presentation:**

This report describes a case of RAH in TSC in an elderly patient. The patient was a 62-year-old woman and experienced pain in the lower left abdomen for two years. Bilateral renal angiomyolipoma, multiple hepatic angiomyolipoma and multiple pulmonary nodules were demonstrated using computed tomography (CT). Brain CT showed bilateral multiple calcification near by the cella lateralis. A clinical diagnosis of TSC was made. Visual acuity (decimal) in the right and left eye was determined to be 0.6 and 0.8, respectively. SD-OCT revealed a retinal tumour in the inner layer of the retina in the right fundus and a pre-retinal membrane which may have evolved later.

**Conclusions:**

A routine ophthalmic examination is advised for patients suspected of having TSC to prevent this condition from being overlooked. In addition, an OCT examination can be used to predict the development and likelihood of RAH.

## Background

Tuberous sclerosis complex (TSC) is an autosomal dominant disorder that causes benign tumours in multiple organs [1]. Retinal astrocytic hamartoma (RAH) occurs in 40–50% of patients with TSC [2]. RAH, which is relatively static with little potential for aggressive behaviour, is largely characterised by benign tumours, and astrocyte enlargement and proliferation in the retina [3]. Generally, TSC occurs in children or young patients, and rarely affects the elderly. There have been a number of reports in the literature on RAH in TSC in young patients. Here, a case of RAH in TSC in an elderly female patient is reported.

## Case presentation

A 62-year-old woman presented at our institution, having experienced pain in the lower left abdomen for two years. She was admitted to the urology unit at the hospital, and underwent a routine ophthalmic examination. She had a history of hypertension (10 years). The patient had no ophthalmological symptoms. Visual acuity (decimal) in the right and left eye was determined to be 0.6 and 0.8, respectively. The manifest refraction was + 1.50DS/− 0.75 DC× 100° in the right eye and + 1.00DS in the left eye.

**Fig. 1:**
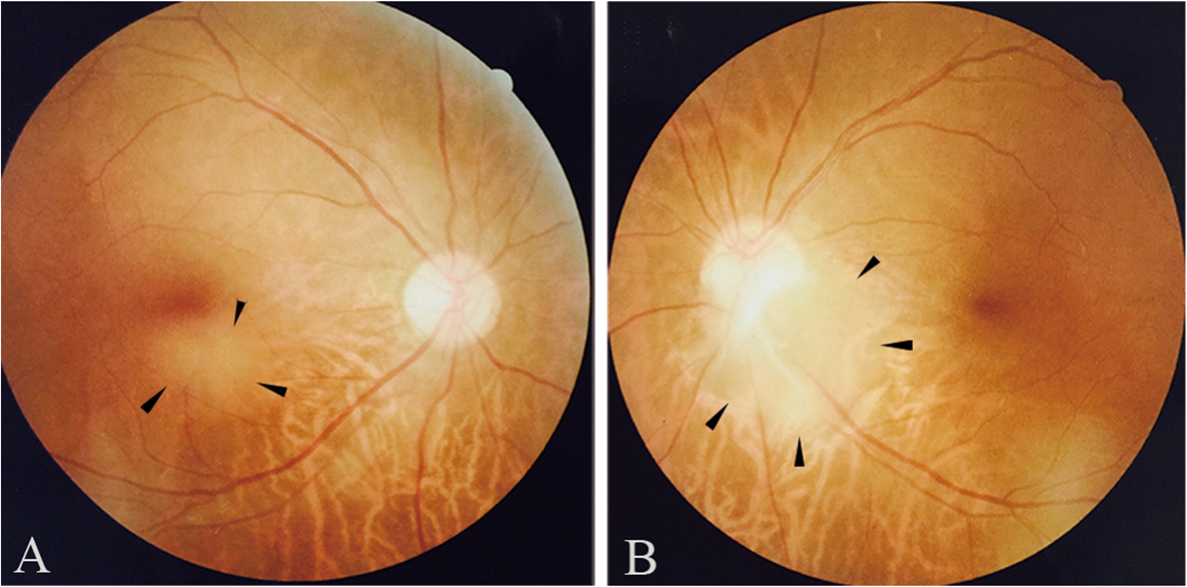
**a** Fundus photograph revealed an astrocytic hamartoma located at the inferior macular in the right eye. **b** Fundus photograph revealed an astrocytic hamartoma located at the subtemporal rim of the optic disc in the left eye

**Fig. 2:**
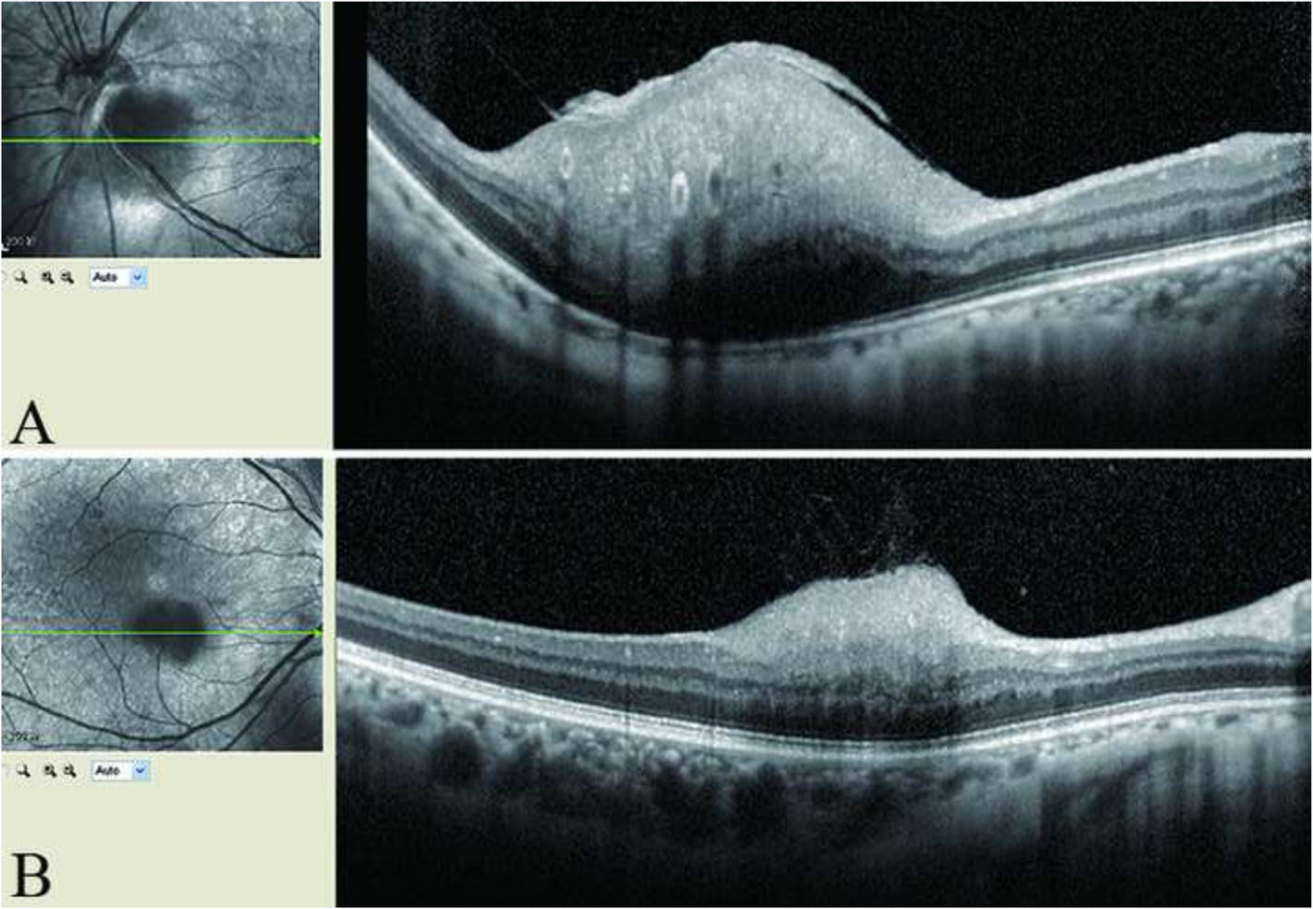
**a** SD-OCT revealed a retinal tumor in the inner layers of the retina in the right fundus. **b** SD-OCT showed epiretinal membrane and a retinal tumor in the inner layers of the retina in the left fundus

The anterior segment examination results were normal. Ophthalmoscopy revealed RAH located at the inferior macular fovea in the right fundus (Fig. 1a) and at the subtemporal rim of the optic disc in the left fundus (Fig. 1b). Spectral domain optical coherence tomography (SD-OCT) revealed a retinal tumour in the inner layer of the retina in the right fundus (Fig. 2a). OCT demonstrated a pre-retinal membrane and retinal tumour located in the inner layer of the retina in the left fundus (Fig. 2b). Adenoma sebaceum was seen in the *ala nasi,* with no ash-leaf spots. Computed tomography (CT) revealed bilateral renal angiomyolipoma, multiple hepatic angiomyolipoma and multiple pulmonary nodules. Brain CT showed extensive bilateral calcification near the *cella lateralis.* The values obtained for the patient’s serum albumin and haemoglobin were 36.7 g/L and 99 g/L, respectively. The decreased serum albumin and haemoglobin levels may have been due to renal dysfunction affected by bilateral renal angiomyolipoma. The laboratory tests (erythrocyte sedimentation rate, C-reactive protein, tuberculin, syphilis, human immunodeficiency virus and hepatitis) were normal. The patient did not consent to an aspiration biopsy of renal angiomyolipoma. A clinical diagnosis of TSC was made. Everolimus was prescribed to the patient to improve abnormalities of the kidney.

## Discussion and conclusions

TSC was first clearly documented by Bourneville in 1880, and was shown to involve multiple organs, including the skin, brain, lungs, eyes, heart, kidneys and bones [4]. RAH is the primary finding in TSC in the retina, and occurs in approximately 50% of patients [5]. A finding of more than one RAH is considered to be significant and specific enough to constitute a major feature of TSC [6]. TSC, which is relative static with little potential for aggressive behaviour, is characterised by benign tumours, and astrocyte enlargement and proliferation in the retina. In rare cases, the tumour enlarges progressively, leading to complications such as exudative retinal detachment, vitreous haemorrhage and neovascular glaucoma [7].

Generally, TSC occurs in infants or children, and rarely affects adults. Barbullushi et al. described the first case of TSC in an elderly patient in 2002, and, since then, only a few reports have been published of cases of TSC in elderly patients [8]. The patient, in our case, was a 62-year-old woman with no ocular symptoms. Fundus photography revealed an RAH located at the bilateral fundus (Fig. 1). OCT also demonstrated a retinal tumour in the inner layer of the retina in both fundi (Fig. 2). The patient also presented with bilateral renal angiomyolipoma, multiple hepatic angiomyolipoma and multiple pulmonary nodules. A clinical diagnosis of TSC was made using TSC diagnostic criteria [6]. RAH should not be confused with other conditions. Important differential diagnoses that need to be excluded are retinoblastoma and choroidal melanoma, as well as several other ophthalmical conditions in order to avoid unnecessary enucleation [5]. The current patient was treated with everolimus to improve abnormalities of the kidney.

In 2016, Pichi et al. classified RAH into four types, based on the OCT findings [9]. This classification was later modified by Mutolo et al., who divided type II into two groups; type IIa and type IIb. The modifications were calculated according to the height of the tumour and intratumoural appearance [10]. Kato et al. proposed that the classification of RAH should include systemic manifestations [11]. In the present case, the elevated tumour, in conjunction with internal retinal disorganisation and mild, minimal vitreoretinal adherence in both the patients’ eyes, was consistent with a type IIb classification of RAH. Elsewhere, it was reported that retinal disorganisation was limited to the inner retina in 20%, outer retina in 0% and full retina in 33% of cases when RAH was located within the retinal nerve fibre layer [12]. The author also highlighted the characteristic “moth-eaten” spaces and posterior shadowing identified using OCT. Retinal disorganisation in our patient was limited to the inner retina. “Moth-eaten” spaces were visible in the left eye.

Performing an ophthalmical examination is often neglected because pain is reported in other parts of the body. Our patient was referred to us following regular interdisciplinary consultations at our institution. A thick pre-retinal membrane with evidence of traction on the surface of the RAH was identified using OCT (Fig. 2b). It is possible that this may have developed later. The elderly patient in the present case study needs to be followed-up regularly to monitor and identify any possible changes timeously.

In summary, TSC is an autosomal dominant disorder that is associated with the development of benign tumours in multiple organs. RAH is a major feature of TSC in the retina. RAH needs to be differentially diagnosed from choroidal melanoma in order to avoid unnecessary enucleation in adult cases. The first consulted physician should inculcate the importance of routine ophthalmical examinations in patients to prevent a TSC diagnosis from being overlooked. It is proposed that OCT should be used to predict the development and likelihood of RAH occurring.

## Abbreviations

CT: Computed tomography
SD-OCT: Spectral domain optical coherence tomography
TSC: Tuberous sclerosis complex
